# Association between poor oral health and diabetes among Indian adult population: potential for integration with NCDs

**DOI:** 10.1186/s12903-019-0884-4

**Published:** 2019-08-20

**Authors:** Ishita Rawal, Shreeparna Ghosh, Safraj Shahul Hameed, Roopa Shivashankar, Vamadevan S. Ajay, Shivani Anil Patel, Michael Goodman, Mohammed K. Ali, K. M. Venkat Narayan, Nikhil Tandon, Dorairaj Prabhakaran

**Affiliations:** 1 Centre for Chronic Disease Control, C-1/52, 2nd Floor, Safdarjung Development Area, New Delhi, 110016 India; 2 Immunization Technical Support Unit (ITSU-MoHFW), B-28 Qutub Institutional Area (first floor), New Delhi, India; 3 Hubert Department of Global Health, Emory University, 1518 Clifton Rd NE, Atlanta, GA USA; 4 Emory University Rollins School of Public Health, 1518 Clifton Road, NE CNR 3021, Atlanta, GA USA; 5 Department of Endocrinology and Metabolism, All India Institute of Medical Sciences, Ansari Nagar (East), New Delhi, 110029 India; 6 Public Health Foundation of India, Plot No. 47, Sector 44, Gurgaon, 122002 India; 7 Department of Epidemiology, London School of Hygiene and Tropical Medicine, London, UK

**Keywords:** Dental caries, Periodontitis, Diabetes, Non-communicable diseases, Low- and middle-income countries

## Abstract

**Background:**

Studies in high-income countries have reported associations between oral health and diabetes. There is however a lack of evidence on this association from low and middle-income countries, especially India. The current study aimed to assess the prevalence of common oral diseases and their association with diabetes.

**Methods:**

This cross-sectional study was nested within the second Cardiometabolic Risk Reduction in South Asia Surveillance Study. A subset of study participants residing in Delhi were administered the World Health Organization’s Oral Health Assessment Questionnaire and underwent oral examination for caries experience and periodontal health assessment using standard indices. Diabetes status was ascertained by fasting blood glucose, glycosylated hemoglobin values or self-reported medication use. Information was captured on co-variates of interest. The association between oral health and diabetes was investigated using Multivariable Zero-Inflated Poisson (ZIP) regression analysis.

**Results:**

Out of 2045 participants, 47% were women and the mean age of study participants was 42.17 (12.8) years. The age-standardised prevalence (95% confidence interval) estimates were 78.9% (75.6–81.7) for dental caries, 35.9% (32.3–39.6) for periodontitis. Nearly 85% participants suffered from at least one oral disease. Compared to diabetes-free counterparts, participants with diabetes had more severe caries experience [Mean Count Ratio (MCR) = 1.07 (1.03–1.12)] and attachment loss [MCR = 1.10 (1.04–1.17)]. Also, the adjusted prevalence of periodontitis was significantly higher among participants with diabetes [42.3%(40.0–45.0)] compared to those without diabetes [31.3%(30.3–32.2)].

**Conclusion:**

We found that eight out of ten participants in urban Delhi suffered from some form of oral disease and participants with diabetes had worse oral health. This highlights the need for public health strategies to integrate oral health within the existing Non-Communicable Disease control programs.

**Electronic supplementary material:**

The online version of this article (10.1186/s12903-019-0884-4) contains supplementary material, which is available to authorized users.

## Background

Oral conditions are known to affect almost half of the world’s population. Dental decay alone affects nearly 2.5 billion people, making it the most prevalent condition worldwide [1]. More than 7% of the world population suffers from severe chronic periodontitis [1]. More Indians suffer from caries and periodontitis than their South Asian counterparts [2]. A recent survey reports prevalence of caries and periodontitis among rural Indian adults was nearly 65% for both conditions [3]. It has been estimated that the total health loss associated with oral conditions is comparable to that for hypertensive heart disease, schizophrenia, and all maternal conditions combined [1]. Besides, India has the maximum number of adults with diabetes in the South-East Asian Region (72.9 million) with the numbers expected to rise to 134 million in 2045 [4].

Epidemiological studies indicate that there are several oral manifestations of diabetes such as periodontitis, dry mouth, root caries, candidiasis [5]. There are pathways linking diabetes with oral diseases, especially periodontitis, which is often referred to as the sixth complication of diabetes [6]. One of the first studies to conclude an association between periodontitis and diabetes was conducted among Pima Indians [7]. The risk of periodontitis was threefold among those with diabetes when compared to those without.

Oral health problems are associated with pain, compromised mastication, xerostomia that profoundly affect overall quality of life, loss of work hours and are often expensive to treat [8, 9]. Despite this, oral health remains an under-recognized and neglected global health issue. Given the burden of oral diseases and their association with diabetes, there is a strong pathophysiological basis for addressing oral health problems within general healthcare practice [10, 11]. Such a comprehensive approach may be particularly useful in Low and Middle-Income Countries (LMICs) for judicious use of resources and reducing the associated expenditure. Despite these considerations, the national Non-Communicable Disease (NCD) programs in most LMICs do not encompass oral health, and data on the co-occurrence of oral diseases with NCDs are scant. To address this knowledge gap, we planned to assess the prevalence of oral diseases and their association with diabetes in a subset of participants enrolled in a population-based cohort in India.

## Methods

### Study participants

The Cardio-metabolic Risk Reduction in South Asia (CARRS) Study Cohort 2 is a population-based study conducted in Chennai and New Delhi, India and in Karachi, Pakistan. The CARRS Oral Health sub-study was nested within the baseline assessment of the Delhi site (2014). A representative sample of adults ≥20 years of age was identified using multistage cluster sampling. This scheme follows the protocol of an earlier study (CARRS − 1), described elsewhere [12]. The primary sampling unit in Delhi were the municipal wards, from which, census enumeration blocks (five per ward) and households (*n* = 25 per block) were randomly selected in that order. Two participants were approached from each household to participate. A subset of CARRS participants was randomly selected for this sub-study and written informed consent was obtained.

**Fig. 1:**
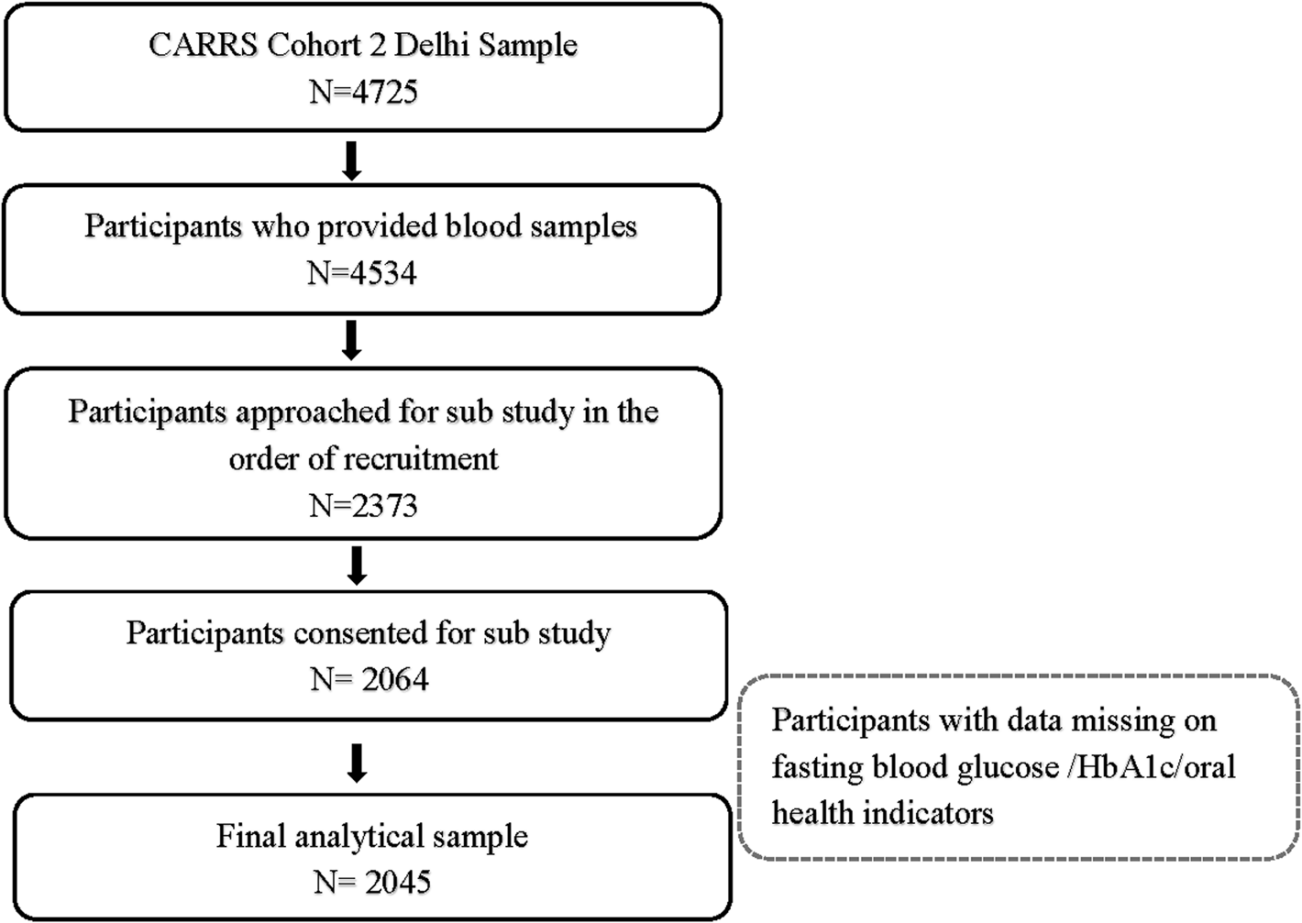
Participant recruitment flowchart

The minimum sample size (*n* = 1965) required for the sub-study was calculated using prevalence estimates of periodontitis from a previous community based study with 5% absolute error [13]. However, more participants were recruited to account for any possible missing information on diabetes status. Figure 1 outlines the recruitment flowchart for this study.

Ethical approval was obtained from the Institutional Review Board of Public Health Foundation of India, New Delhi.

### Study procedures

#### Oral health assessment

Oral health was assessed through a combination of interviewer-administered questionnaire and clinical examination performed by qualified dentists assisted by trained field personnel at neighborhood locations accessible to the participants. Five dentists attended a three-day training in study methods. Calibration sessions included interpretation of scores from Decayed, Missing, Filled Tooth (DMFT) and Loss of Attachment (LOA) Indices, the average intraclass correlation and kappa statistic were 0.97 (0.94–0.99) and 0.71 (0.58–0.83) respectively. Data was collected using a tablet and took 25–30 min per participant. The application was developed using an open-source software, open data kit, with appropriate logic and range checks that enabled real time transfer [14].

World Health Organization’s Oral Health Assessment Questionnaire was used to capture information on oral hygiene practices, self-reported oral health problems, and service utilisation [15].

The examination began with evaluation and palpation of extraoral features for ulcers, erosion or fissures on face, neck, commissures and vermilion border or enlarged lymph nodes.

For intraoral examination, both hard and soft tissues were examined, measures outlined in Table 1. The oral cavity was divided into four quadrants; maxillary (upper) right-left and mandibular (lower) left-right, examined in that order from anterior to posterior direction. The overall caries experience was recorded using the standardised DMFT index and prevalence of dental caries was assessed using the Decayed component of the index. Gingival and periodontal health was assessed using Community Periodontal Index (CPI) Loss of Attachment (LOA) index. Unlike DMFT, periodontal examination was done by assessment of six index teeth (representative teeth from each sextant: right-left maxillary/mandibular posterior and maxillary/mandibular anterior). Both, CPI and LOA were used for assessment of gingival and periodontal health. Prevalence of periodontitis was measured according to the U.S. Centres for Disease Control and Prevention and the American Academy of Periodontology [16].

**Table 1:** Clinical assessment measures of oral health status

Examination	Purpose and Index	Instrument Used
EXTRAORAL ASSESSMENT	Assessment of extraoral abnormalities	Visual examination and palpation
INTRAORAL ASSESSMENT	Assessment of both, hard and soft tissues	
Dentition	Assessment of teeth	
Caries experience	Tooth decay, loss and restoration DMFT Index	Mouth mirror, curved double ended probe
Fluorosis	Fluorosis Dean’s index	
Traumatic dental injuries	Trauma	
Soft tissue	Assessment of tooth supporting structure and oral mucosa	
Gingivitis	Status of gingiva CPI Index	Mouth mirror, WHO CPI Probe
Periodontitis	Status of periodontium and attachment loss LOA Index	
Oral mucosal lesions	Pre-malignant, malignant and other conditions of the mucosa	Visual examination and palpation

*DMFT* Decayed Missing Filled Teeth

*LOA* Loss of attachment Index

*WHO* World Health Organisation

#### Socio-demographic factors

The covariates considered were based on evidence of their role in the association between diabetes and oral health outcomes. Data for these were obtained from CARRS-2 cohort [12]. Age was categorized as 20–39, 40–59 and ≥ 60 years. With respect to monthly household income [in Indian Rupee (INR)], the study population was divided into four groups; < 10,000, 10,001–20,000, 20,001–30,000, and ≥ 30,001. Education status was classified as primary school or less, high school, secondary to intermediary school, or graduate degree and above. The summary household asset index variable was developed based on the principal component analysis, took into consideration type of kitchen, drinking water source, type of toilet facility, and ownership of a refrigerator, washing machine, microwave, grinder, digital video disc player, computer, car, motorcycle and bicycle. For the purposes of the current analysis, the distribution of the household asset index was divided into tertiles (low, medium, high).

#### Diabetes and modifiable risk factors

Diabetes was defined as: measured fasting plasma glucose ≥126 mg/dl and/or HbA1c ≥6.5% or receipt of anti-diabetes medications [17]. Participants were interviewed about ‘ever-use’ of tobacco (both smoking and smokeless forms) and alcohol. Dietary habits were ascertained using a food frequency questionnaire, adapted from the INTERHEART study, administered to obtain information on usual intake of fruits, desserts and sugary beverages (each expressed as daily/weekly, monthly, never/less than once a month) [12, 18]. Body Mass Index (BMI) was calculated from the measured values of weight and height and classified as underweight (< 18.5 kg/m^2^), normal (18.5 to 24.9 kg/m^2^), overweight (25.0 to 29.9 kg/m^2^) or obese (≥30.0 kg/m^2^).

### Statistical methods

Participant characteristics were presented as means and standard deviations (SD) for continuous variables and as percentages for categorical variables. The two main outcome variables for measuring oral health status were DMFT for caries experience and LOA scores for attachment loss (both count variables). Many participants had zero observations for these scores therefore Zero-Inflated Poisson (ZIP) regression was used. ZIP regression generates two separate models. The first is a logit model that estimates the likelihood of having a DMFT/LOA score of zero (vs. any other value) and generates the corresponding odds ratio (OR) along with the 95% confidence interval (CI). The second is a Poisson model that predicts severity of the oral condition among those who had scores of > 0; the results of this second model are expressed as mean count ratios (MCR) and 95% CIs [19]. The final model has been adjusted for the following variables: age, gender, educational status, income, asset index, frequency of cleaning, service utilisation, consumption of fruits, deserts, sugary drinks, tobacco and alcohol consumption and BMI. Population attributable fraction (PAF) of diabetes and common modifiable risk factors were also estimated (Additional file 1: Appendix, Box 1).

As the CARRS-Oral Health study included only a subset of the CARRS-2 cohort participants, key background characteristics of all participants were included in a separate regression model to calculate the probabilities of selection into the sub-study. Inverse values of these probabilities were used as weights in the sensitivity analyses to account for potential selection bias (Additional file 1: Appendix, Table S1).

To ensure that the sub-study sample remained representative of the Delhi target population, final analysis weight was computed by multiplying the sub-study selection weight by the overall CARRS-2 survey weight. Analysis was performed using Stata v14.2 (StataCorp, College Station, Texas, USA).

## Results

Out of 2045 participants, 47% were women. Mean (SD) age of the participants was 42.17 (12.8) years. Around 27% participants reported ever-use of tobacco (men 46%, women 7%) and 26% had ever-consumed alcohol (men 48%, women 1%). Average (SD) BMI was 25.91 (5.1) kg/m^2^, fasting plasma glucose was 107.69 (40.4) mg/dl, HbA1c was 5.90 (1.2) % and 16.9 participants had diabetes. Table 2 shows the background characteristics of study population.

**Table 2:** Background characteristics of study participants and oral health scores (*N* = 2045)

Characteristics	Total *N* (%)	DMFT score Mean (SD)	LOA score^a^ Mean (SD)
SOCIO-DEMOGRAPHIC FACTORS			
Age group, in years			
20–39	814 (46.0)	4.61 (3.6)	0.11 (0.3)
40–59	929 (41.5)	6.00 (5.4)	0.23 (0.2)
≥ 60	302 (12.6)	13.07 (10.7)	0.50 (0.6)
Gender			
Men	929 (52.8)	5.76 (5.8)	0.23 (0.4)
Women	1116 (47.2)	6.78 (6.4)	0.17 (0.3)
Education status			
Graduate and above	531 (27.9)	5.36 (5.8)	0.99 (0.3)
Higher secondary	573 (26.5)	6.25 (6.4)	0.18 (0.3)
High school	530 (26.5)	6.37 (5.8)	0.25 (0.4)
Primary or below	411 (19.1)	7.38 (6.4)	0.32 (0.5)
Monthly household income, in INR			
≤ 10,000	738 (33.8)	6.19 (6.1)	0.26 (0.4)
10,001–20,000	567 (28.6)	6.32 (5.6)	0.20 (0.3)
20,001–30,000	289 (14.2)	5.84 (6.7)	0.21 (0.4)
≥ 30,001	406 (23.3)	6.22 (6.0)	0.11 (0.3)
Household asset index			
Low	725 (34.0)	6.27 (5.7)	0.26 (0.4)
Middle	692 (34.8)	6.13 (5.9)	0.22 (0.4)
High	628 (31.2)	6.35 (6.8)	0.12 (0.3)
ORAL HEALTH PRACTICES			
Frequency of cleaning			
Irregular	46 (2.6)	13.35 (11.7)	0.31 (0.4)
Once a day	1458 (72.3)	5.86 (5.6)	0.21 (0.4)
Twice a day	541 (25.1)	6.62 (6.2)	0.18 (0.3)
Dental service utilization			
Never	642 (32.4)	4.04 (4.4)	0.15 (0.3)
< 6 months	299 (13.6)	7.38 (6.9)	0.17 (0.4)
6–24 months	460 (23.6)	7.29 (6.1)	0.25 (0.4)
> 24 months	644 (30.4)	7.27 (6.9)	0.23 (0.4)
MODIFIABLE RISK FACTORS AND DIABETES			
Frequency of fruits intake			
Daily/weekly	1336 (66.3)	6.08 (6.0)	0.17 (0.4)
Monthly	215 (10.5)	6.90 (6.4)	0.26 (0.4)
Never	494 (23.2)	6.41 (6.3)	0.24 (0.4)
Frequency of sugary drinks consumption			
Daily/weekly	588 (31.1)	5.42 (5.4)	0.14 (0.3)
Monthly	336 (16.1)	6.92 (5.9)	0.21 (0.4)
Never	1121 (52.8)	6.53 (6.5)	0.24 (0.4)
Frequency of desserts consumption			
Daily/weekly	682 (36.0)	5.75 (5.5)	0.17 (0.4)
Monthly	767 (37.2)	6.29 (5.9)	0.16 (0.3)
Never	595 (26.8)	6.84 (7.1)	0.29 (0.4)
Tobacco consumption^b^			
Yes	466 (27.5)	5.99 (6.3)	0.32 (0.4)
No	1579 (72.6)	6.34 (5.7)	0.16 (0.3)
Alcohol consumption^b^			
Yes	409 (25.7)	5.75 (5.0)	0.23 (0.3)
No	1636 (74.3)	6.42 (6.5)	0.19 (0.4)
BMI groups			
Underweight	98 (5.4)	3.88 (4.3)	0.19 (0.3)
Normal	804 (41.5)	6.46 (6.0)	0.22 (0.4)
Overweight	695 (32.4)	6.11 (5.9)	0.20 (0.4)
Obese	437 (20.6)	6.58 (6.8)	0.16 (0.3)
Diabetes Status			
Diabetes	406 (16.9)	7.85 (8.6)	0.28 (0.5)
No diabetes	1639 (83.1)	5.92 (5.5)	0.18 (0.4)

*DMFT* Decayed, Missing, Filled Tooth

*LOA* Loss of Attachment

*SD* Standard Deviation

*INR* Indian Rupee

*BMI* Body Mass Index

^a^Mean of mean LOA scores

^b^Ever-used

The age-standardised prevalence (95% CI) estimates were 78.9% (75.6–81.7) for dental caries, 35.9% (32.3–39.6) for periodontitis, 14.9% (10.8–20.1) for fluorosis and 3.1% (2.2–4.3) for dental trauma. Only 15% participants had healthy gingiva, bleeding on probing and calculus deposits were observed in 57 and 24% participants respectively. The mean number of sound teeth present in study participants were 25.75 (6.1). Oral pre-malignant lesions; leucoplakia and lichen planus were provisionally diagnosed in 1.6% of participants. Nearly 85% participants suffered from at least one form of oral disease. With respect to oral hygiene practices, only 26% participants reported cleaning their teeth twice-daily and 31% participants had never visited a dental care facility.

The mean (SD) values for DMFT and LOA indices were 5.71 (5.0) and 0.20 (0.4) respectively. Details of score distributions are presented in Table 2.

Estimates from the logit model of ZIP regression showed that the odds of having zero score for caries experience and attachment loss were significantly higher among younger participants, those with higher education, higher household income and had never visited a dental facility. The likelihood of having zero LOA score was significantly lower among those who had ‘ever-used’ tobacco.

The Poisson component showed that among those with score > 0, participants in older age and low household income groups had significantly greater severity of caries experience and attachment loss when compared to their reference groups (Table 3). Women had higher DMFT, but lower LOA counts relative to men. MCRs were significantly higher among those who did not clean their teeth regularly. Participants with diabetes presented with more severe caries experience and attachment loss compared to diabetes-free participants. The MCR (95% CI) estimates for DMFT was 1.07 (1.03–1.12) and LOA was 1.10 (1.04–1.17) after adjusting for co-variates.

**Table 3:** Multivariable results of Zero Inflated Poisson regression by determinants of oral health (*N* = 1988)

	DMFT		LOA	
	Logit model	Poisson model	Logit model	Poisson model
	OR (95% CI) *n* = 1988	MCR (95% CI) *n* = 1750	OR (95% CI) *n* = 1988	MCR (95% CI) *n* = 710
SOCIO-DEMOGRAPHIC FACTORS				
Age group, in years				
20–39	1	1	1	1
40–59	0.93 (0.67–1.30)	1.20^d^ (1.15–1.26)	0.32^d^ (0.24–0.41)	1.35^d^ (1.22–1.48)
≥ 60	0.35^c^ (0.17–0.69)	2.43^d^ (2.30–2.62)	0.05^d^ (0.03–0.08)	2.65^d^ (2.40–2.93)
Gender				
Men	1	1	1	1
Women	0.70 (0.48–1.03)	1.09^d^ (1.04–1.14)	1.06 (0.80–1.40)	0.89^d^ (0.84–0.95)
Education status				
Graduate and above	1	1	1	1
Higher Secondary	0.80 (0.53–1.21)	1.08^c^ (1.03–1.14)	0.73 (0.53–1.01)	1.07 (0.99–1.16)
High school	0.70 (0.44–1.13)	1.04 (0.98–1.10)	0.51^d^ (0.36–0.72)	1.03 (0.95–1.12)
Primary or below	0.64 (0.36–1.14)	1.02 (0.95–1.08)	0.58^c^ (0.39–0.87)	1.06 (0.97–1.17)
Monthly Household income, in INR				
≥ 30,001	1	1	1	1
≤ 10,000	0.73 (0.42–1.27)	1.17^d^ (1.10–1.25)	0.55^d^ (0.37–0.83)	1.13^b^ (1.03–1.25)
10,001–20,000	0.68 (0.41–1.14)	1.07^b^ (1.01–1.13)	0.63^b^ (0.44–0.91)	0.96 (0.88–1.04)
20,001–30,000	0.84 (0.50–1.39)	1.07^b^ (1.01–1.13)	0.69 (0.47–1.01)	1.09 (1.00–1.19)
Household asset index				
High	1	1	1	1
Low	0.79 (0.42–1.38)	0.98 (0.92–1.05)	0.75 (0.50–1.12)	0.93 (0.84–1.02)
Middle	0.92 (0.59–1.42)	0.95 (0.91–1.00)	1.03 (0.75–1.42)	0.97 (0.91–1.05)
ORAL HEALTH PRACTICES				
Frequency of cleaning				
Twice a day	1	1	1	1
Irregular	0.27 (0.06–1.22)	1.68^d^ (1.53–1.83)	0.73 (0.35–1.52)	1.94^d^ (1.74–2.16)
Once a day	0.89 (0.63–1.25)	0.98 (0.95–1.02)	1.24 (0.97–1.58)	1.08^c^ (1.02–1.14)
Dental service utilization				
< 6 months	1	1	1	1
6–24 months	1.11 (0.59–2.09)	0.97 (0.97–1.07)	0.93 (0.66–1.31)	0.89^d^ (0.82–0.97)
> 24 months	1.31^d^ (0.73–2.37)	1.02 (0.97–1.07)	1.15 (0.83–1.59)	0.99 (0.92–1.06)
Never	4.71^d^ (2.70–8.18)	0.72^d^ (0.67–0.76)	2.14^d^ (1.51–3.04)	0.74^d^ (0.67–0.81)
MODIFIABLE RISK FACTORS AND DIABETES				
Frequency of fruits intake				
Daily/weekly	1	1	1	1
Monthly	0.71 (0.41–1.21)	1.10^c^ (1.04–1.16)	0.69^b^ (0.48–0.99)	1.09^b^ (1.01–1.18)
Never	0.81 (0.55–1.18)	1.06^c^ (1.02–1.11)	0.86 (0.65–1.12)	1.07^b^ (1.01–1.14)
Frequency of sugary drinks consumption				
Never	1	1	1	1
Daily/weekly	0.91 (0.64–1.29)	1.00 (0.96–1.04)	1.10 (0.85–1.42)	1.03 (0.96–1.10)
Monthly	0.89 (0.58–1.38)	1.11^d^ (1.06–1.16)	0.89 (0.65–1.20)	1.00 (0.93–1.08)
Frequency of desserts consumption				
Never	1	1	1	1
Daily/weekly	0.79 (0.53–1.18)	1.02 (0.97–1.07)	1.30 (0.98–1.74)	1.09^b^ (1.02–1.17)
Monthly	0.82 (0.56–1.20)	1.02 (0.97–1.06)	1.39^b^ (1.06–1.83)	0.97 (0.91–1.03)
Tobacco consumption^a^				
No	1	1	1	1
Yes	1.35 (0.89–2.03)	1.05 (1.00–1.10)	0.69^b^ (0.51–0.94)	0.95 (0.88–1.02)
Alcohol consumption^a^				
No	1	1	1	1
Yes	0.84 (0.55–1.28)	0.97 (0.91–1.02)	1.00 (0.72–1.38)	0.83^d^ (0.77–0.90)
BMI groups				
Normal	1	1	1	1
Underweight	1.24 (0.68–2.27)	0.91^b^ (0.82–0.99)	1.14 (0.67–1.94)	0.92 (0.79–1.08)
Overweight	0.73 (0.50–1.04)	0.92^d^ (0.89–0.97)	0.97 (0.75–1.26)	1.03 (0.96–1.09)
Obese	0.82 (0.52–1.27)	0.99 (0.94–1.05)	1.21 (0.88–1.67)	1.24^d^ (1.15–1.34)
Diabetes Status				
No Diabetes	1	1	1	1
Diabetes	0.95 (0.62–1.45)	1.07^c^ (1.03–1.12)	0.90 (0.68–1.19)	1.10^c^ (1.04–1.17)

*DMFT* Decayed, Missing, Filled Tooth Score

*LOA* Loss of Attachment Score

*OR* Odds Ratio

*CI* Confidence Intervals

*MCR* Mean Count Ratio

*INR* Indian Rupee

*BMI* Body Mass Index

^a^Ever used

^b^*p* < 0.05

^c^*p* < 0.01

^d^*p* < 0.001

**Fig. 2:**
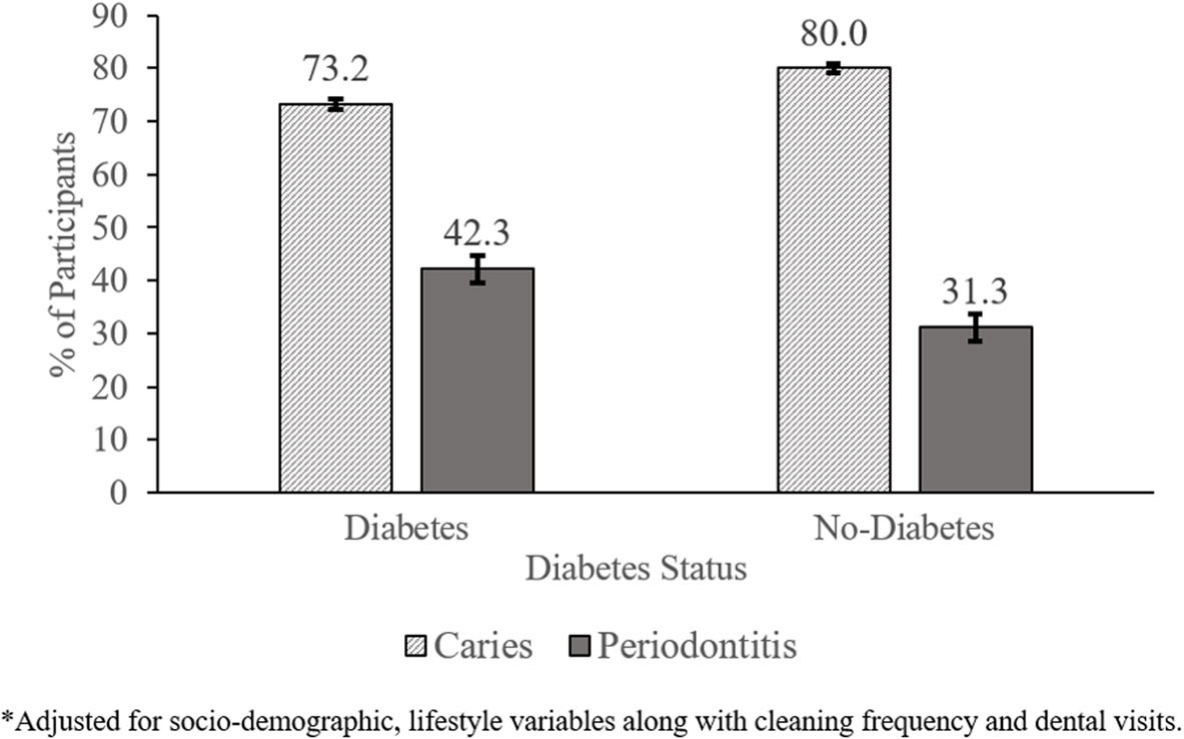
Adjusted* prevalence of dental caries and periodontitis by diabetes status

Figure 2 shows that the adjusted prevalence of periodontitis was significantly higher among participants with diabetes 42.3 (95%CI: 40.0–45.0) compared to their disease-free counterparts 31.3 (30.3–32.2) but prevalence of caries was higher among those without diabetes [80.1 (79.7–80.4) vs. 73.2 (72.3–74.1)]. Mean number of sound teeth present among those with diabetes was significantly lower than those without diabetes (24.14 vs. 26.07; *p* < 0.001). Collectively, diabetes and common risk factors (diet, tobacco use, frequency of cleaning) accounted for 12% & 15% of the PAF of DMFT and LOA scores respectively (Additional file 1: Appendix, Table S2).

## Discussion

We found a high prevalence of dental caries and periodontitis among Delhi CARRS-cohort members. These results are consistent with other studies reporting prevalence estimates from India [20]. Previous studies also confirm a similar service utilization pattern, with 77% participants ‘ever-visiting’ a dental facility [21]. Study participants who had ‘never-visited’ a dental facility had greater likelihood of having zero scores. Good oral health could be the reason for their non-visits as oral health is often perceived unimportant and visits are associated with symptoms such as pain or discomfort [22].

Most of the participants practiced inadequate oral hygiene. This results in improper removal of dental plaque and debris which adheres to the tooth surface and gingiva leading to caries and gingival inflammation. A similar inflammatory response is observed in tobacco users resulting in gingival damage [23]. This could be the reason for lower odds of zero score among tobacco users in the study. Though sugar-rich diet is an important risk factor for caries but our data did not confirm this association [24]. Several others factors, that include: nature and adhesiveness of diet, salivary characteristics, masticatory functions, and nutritional inadequacy of micronutrients are also accountable [24]. These details were not captured in our study.

Our finding, that participants with diabetes had poor periodontal health than those without diabetes, corresponds with studies from high income countries [7, 25–27]. The National Health and Nutrition Examination Survey III found that adults with HbA1c > 9% had a significantly higher prevalence of severe periodontitis than those without diabetes [OR = 2.90 (1.40–6.03)] after controlling for confounders [27]. Studies also report participants with diabetes had greater tooth loss and gingival bleeding on probing [26]. Individuals with uncontrolled diabetes also have a higher risk of infection, as well as prolonged healing time [28]. We found lower prevalence of caries in participants with diabetes but higher cumulative DMFT score compared to those without diabetes. This could be attributed to greater missing-tooth component [29].

Studies on the cooccurrence of dental caries and diabetes have yielded non-conclusive results [30, 31]. Diet modification is often advised for patients with diabetes. The restricted consumption of carbohydrates may have a non-cariogenic effect on the participants with diabetes in the study additionally the anti-microbial defense capacity of saliva is not weakened in diabetes [32]. On the contrary, increased salivary glucose concentration could be the reason for increased caries among patients with diabetes.

Mechanistic links suggest Hyperglycaemia often results in altered cellular immunity, proliferation of bacteria, and formation of advanced glycation end-products (AGEs) [5]. Altered cellular immunity results in dysfunction of cells, inflammation and degradation of supporting connective tissue [6]. Bacterial proliferation also exacerbates the inflammatory response contributing to periodontal destruction [4]. Excess glucose forms AGEs on coming in contact with structural proteins [33, 34]. These end-products stimulate endothelial receptors and perpetuate a series of inflammatory events by attracting monocytes and ultimately leading to degradation of the attachment apparatus. Furthermore, hyperglycaemic environment impairs the function of fibroblasts and predisposes collagen to degradation by matrix metalloproteinase enzymes, thereby preventing tissue repair and regeneration [35]. Reduced salivary flow, pH, dryness of mouth and gingival recession is also commonly observed in diabetes, predisposing to dental decay and tooth loss [36]. Previous studies have concluded that diabetes has many adverse effects on periodontal tissue, and conversely, periodontitis may aggravate the hyperglyceamia. Combined results from 13 meta-analyses indicate a significant but small effect of periodontal treatment on improved HbA1c in diabetes patients [28]. However, it warrants additional high-quality studies to obtain a conclusive evidence.

Oral health care is conventionally disease-oriented, curative in nature and serves limited people due to high costs. Given the burden of unmet dental care needs and their association with systemic conditions, it is imperative to bridge the disconnect and re-orient oral-health services to be integrated and prevention-based at community level. It is propagated that these services should primarily be based on education to increase awareness and influence attitude of the people in seeking oral health care [37]. Pilot projects have demonstrated positive outcomes where health-facility staff was trained to deliver oral health education messages by integrating within their primary health care tasks [37]. Better collaboration by minimal training and expanding the role of other health professionals can be useful in providing basic oral health education and early recognition of oral conditions for further referral. Integration of oral health with the national chronic disease programs require serious consideration given the high burden of oral diseases, NCDs and shared set of risk factors [10]. The Government of India’s National Programme for Prevention and Control of Cancer, Diabetes, Cardiovascular Disease and Stroke (NPCDCS) launched in 2010 lacks any oral health goals within it [38]. However, its recent expansion includes screening, early detection, management and referral of cases of three common cancers, oral cancer being one of them [39]. Therefore, an integrated approach inclusive of oral health may have greater benefits for health of the community and facilitate judicious use of resources.

Our study is among the few studies in India to have followed a comprehensive approach by integrating oral health with NCDs [40–43]. To our knowledge, this is the first study with the above strategy using a representative sample from community. We have used standard survey methods that will enable comparison of findings from other studies. Although index teeth examination may underestimate the prevalence of periodontal disease, this method is efficient in estimating the mean periodontal scores. Adoption of this method prevented examiner fatigue and excessive participant time. Other limitations include the fact that clinical examination was not supported by radiographic or saliva sample investigations, cross-sectional nature limits conclusion of causal relations and duration and severity of diabetes was not used in present analysis.

## Conclusion

In summary, we found that eight out of ten participants in urban Delhi suffered from some form of oral disease and participants with diabetes had worse oral health. The high burden of oral diseases and diabetes among Indians, evidence of their association and role of shared risk factors provides an opportunity to expand the scope of existing national NCD program by integrating oral health interventions.

## Additional file

Additional file 1: **Appendix.** The file contains Box 1: formulae for calculating population attributable fraction (PAF), **Table S1.** Sample distribution of participants in oral health sub-study and CARRS-2 Delhi cohort and **Table S2.** Population attributable fraction associated with common risk factors. (DOCX 26 kb)

## Data Availability

The datasets used and/or analysed during the current study are available from the corresponding author on reasonable request.

## Abbreviations

AGEs: Advanced glycation end-products
BMI: Body Mass Index
CARRS: Cardio-metabolic Risk Reduction in South Asia
CI: Confidence interval
CPI: Community Periodontal Index
DMFT: Decayed, Missing, Filled Tooth
INR: Indian Rupee
LMIC: Low and Middle-Income Countries
LOA: Loss of Attachment
MCR: Mean Count Ratio
NCD: Non-Communicable Disease
PAF: Population attributable fraction
SD: Standard Deviation
ZIP: Zero-Inflated Poisson
