# Supplementary material for: Association between poor oral health and diabetes among Indian adult population: potential for integration with NCDs
Source: BMC Oral Health. 2019 Aug 20;19:191. doi: 10.1186/s12903-019-0884-4 (PMC6701092; doi:10.1186/s12903-019-0884-4)
Supplement: Supplementary file 1 — Appendix. The file contains Box 1: formulae for calculating population attributable fraction (PAF), Table S1. Sample distribution of participants in oral health sub-study and CARRS-2 Delhi cohort and Table S2. Population attributable fraction associated with common risk factors. (DOCX 26 kb) [file 12903_2019_884_MOESM1_ESM.docx]

**Additional file**

**Association between poor oral health and diabetes among Indian adult population: Potential for integration with NCDs**

Box 1. Formulae for calculating population attributable fraction (PAF)

| 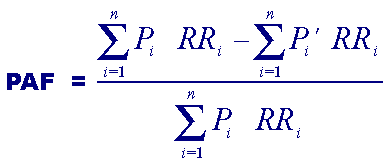   - PAF=Population attributable fraction - Pi = proportion of population at exposure level i, current exposure - P'i = proportion of population at exposure level i, counterfactual or ideal level of exposure - RR = the relative risk ratio at exposure level i - n = the number of exposure levels |
| --- |

Table S1. Sample distribution of participants in oral health sub-study and CARRS-2 Delhi cohort

|  | **CARRS-2 Delhi sample**  (N=4534) | **Sub study analytical sample**  (N=2045) | **Excluded sample**  (N=2489) | **Weighted sub study analytical sample** |
| --- | --- | --- | --- | --- |
| **Age** in years, mean (SD) | 42.0 (13.2) | 41.5 (12.8) | 42.4 (13.5) | 42.2 (13.4) |
| **Age group,** (%) |  |  |  |  |
| 20-39 | 46.3 | 47.3 | 45.5 | 46.0 |
| 40-59 | 41.2 | 42.3 | 40.4 | 41.5 |
| ≥60 | 12.5 | 10.4 | 14.1 | 12.6 |
| **Gender,** (%) |  |  |  |  |
| Men | 53.0 | 50.7 | 54.8 | 52.8 |
| Women | 47.0 | 49.3 | 45.2 | 47.2 |
| **Education status,** (%) |  |  |  |  |
| Graduate and above | 28.9 | 26.5 | 30.6 | 27.9 |
| Higher secondary | 27.1 | 28.1 | 26.3 | 26.5 |
| High school | 24.8 | 27.8 | 22.6 | 26.5 |
| Primary or below | 19.2 | 17.6 | 20.5 | 19.1 |
| **Monthly household income,** in INR (%) | | | | |
| ≤ 10,000 | 32.2 | 36.5 | 28.9 | 33.8 |
| 10,001-20,000 | 29.0 | 28.5 | 29.5 | 28.6 |
| 20,001-30,000 | 14.7 | 14.0 | 15.3 | 14.2 |
| ≥30,001 | 24.1 | 21.0 | 26.4 | 23.3 |
| **Tobacco consumption^*^**,  (%) | 27.0 | 24.6 | 28.8 | 27.45 |
| **Alcohol consumption^*^**,  (%) | 24.7 | 24.1 | 25.1 | 25.7 |
| **BMI** in kg/m^2^,  mean (SD) | 25.8 (5.1) | 25.9 (5.2) | 25.8 (5.0) | 25.9 (5.3) |
| **Diabetes Status,** (%) |  |  |  |  |
| Diabetes | 16.1 | 15.4 | 16.6 | 16.9 |
| No diabetes | 83.9 | 84.6 | 83.4 | 83.1 |

SD: Standard Deviation

INR: Indian Rupee

BMI: Body Mass Index

^*^Ever-used

**CARRS-2 Delhi sample**: Estimates are sample weighted for the CARR-2 Delhi cohort providing blood sample.

**Sub study analytical sample**: Estimates are sample weighted for oral health sub study participants with complete information on oral health indicators and diabetes status.

**Excluded sample**: Estimates are sample weighted for CARRS-2 Delhi participants not a part of oral health sub study or participants of sub study but missing data on oral health indicators and diabetes.

**Weighted sub study analytical sample**: Estimates for sub study participants adjusted for sample weight and inverse probability weight.

Table S2. Population attributable fraction associated with common risk factors

| **Common risk factors** | **DMFT**  **PAF (95% CI)** | **LOA**  **PAF (95% CI)** |
| --- | --- | --- |
| Gender (Male)* | 5.4 (2.7, 8.0) | -6.8 (-13.9, -0.1) |
| Education (Graduate & above)* | 5.6 (1.0, 10.1) | 18.9 (8.4, 28.2) |
| Income (=>30,000)* | 9.9 (4.7, 14.7) | 17.9 (6.6, 27.9) |
| Fruits (Daily/weekly)* | 3.2 (1.5, 4.9) | 5.7 (1.7, 9.5) |
| Drinks (Never)* | 2.2 (0.1, 4.3) | 0.3 (-4.2, 4.7) |
| Dessert (Never)* | 2.7 (-1.2, 6.4) | -7.3 (-15.7, 0.6) |
| Tobacco use (No)* | 0.5 (-1.3, 2.3) | 2.4 (-1.7, 6.3) |
| Alcohol use (No)* | -0.4 (-2.1, 1.3) | -4.2 (-8.2, -0.4) |
| BMI (Normal)* | -2.0 (-4.9, 0.9) | 3.6 (-3.2, 9.9) |
| Frequency of cleaning (Twice a day)* | 1.9 (-1.9, 5.6) | 3.0 (-5.5, 10.7) |
| Diabetes (No)* | 1.5 (0.4, 2.6) | 3.7 (0.6, 6.7) |

DMFT=Decayed, Missing, Filled Tooth Score

LOA=Loss of Attachment Score

PAF=Population attributable fraction

BMI=Body Mass Index

()*=Reference Category
